# Roles of fibrinolytic factors in the alterations in bone marrow hematopoietic stem/progenitor cells during bone repair

**DOI:** 10.1186/s41232-020-00128-5

**Published:** 2020-09-16

**Authors:** Kiyotaka Okada, Minoru Nishioka, Hiroshi Kaji

**Affiliations:** 1 Department of Arts and Science, Kindai University Faculty of Medicine, Osaka-Sayama, Osaka, 589-8511 Japan; 2 Department of Physiology and Regenerative Medicine, Kindai University Faculty of Medicine, Osaka-Sayama, Osaka, 589-8511 Japan

**Keywords:** Bone regeneration, Fibrinolytic factor, Hematopoietic stem/progenitor cells, Stromal cell-derived factor-1, Transforming growth factor-β

## Abstract

In bone tissues, metabolic turnover through bone resorption by osteoclasts and bone formation by osteoblasts, termed bone remodeling, is strictly controlled and maintains homeostasis. Fibrinolytic factors are expressed in osteoclasts and osteoblasts, and are involved in bone remodeling through bone resorption and formation. The repair/regeneration process after bone injury is divided into the acute inflammatory, repair, and remodeling stages. Osteoblasts, osteoclasts, chondrocytes, and macrophages involved in the bone repair process originate from hematopoietic stem/progenitor cells (HSPCs) and mesenchymal stem cells (MSCs) in the bone marrow. Therefore, stem cells in the bone marrow may be strongly influenced by bone injury. The urokinase-type PA (u-PA)/plasminogen (Plg) system functions in macrophage accumulation/phagocytosis through chemokines in the acute inflammatory stage, and Plg increases blood vessel-related growth factor expression, being involved in vascularization in mice. Plasminogen activator inhivitor-1 (PAI-1) causes bone loss and delayed bone repair through the inhibition of osteoblast differentiation in a drug-induced diabetes model in mice. Plg is considered to induce transforming growth factor-β (TGF-β) production in macrophages in the bone repair process, TGF-β release from the extracellular matrix through the activation of matrix metalloproteinase-9 (MMP-9), and stromal cell-derived factor-1 (SDF-1) expression in endosteal preosteoblasts, leading to the induction of bone marrow HSPCs in mice. Based on the above, establishment of a fibrinolytic factor-targeting method efficiently promoting bone repair/regeneration and fracture healing, and development of a new osteoporosis treatment method and diagnostic marker are awaited.

## Background

In bone tissues, metabolic turnover, termed bone remodeling, takes place actively. By this mechanism, bone resorption by osteoclasts and bone formation by osteoblasts are strictly controlled and maintain homeostasis [1]. When the balance of bone remodeling is broken and bone resorption relatively surpasses bone formation, the bone mineral density decreases, resulting in osteoporosis. In the regulation of bone metabolism, endocrine factors, such as parathyroid hormone, play a central role [2]. Regulation by the nervous system and regulatory mechanisms by humoral factors secreted from other tissues, including skeletal muscle, pancreas, and fat, have also been clarified [3]. Furthermore, bone matrix proteins function in the degradation involved in bone remodeling as proteolytic enzymes, including fibrinolytic system enzymes and matrix metalloproteinases (MMPs) [4].

The repair/regeneration process after bone tissue injury is complex and comprises a multistage process. The bone repair process is divided into 3 stages: (1) the acute inflammatory stage, (2) repair stage, and (3) remodeling stage [5]. In the acute inflammatory stage, inflammatory cells, including neutrophils and macrophages, are induced in the injured region, and release cytokines and growth factors. In the repair stage, vascularization and endochondral ossification occur in the injured region. In the remodeling stage, osteoblasts and osteoclasts are involved in bone formation and resorption, respectively, and repair the injury. In addition, local ischemia, stem cell mobilization, and interaction between inflammatory cells are observed in the acute inflammatory stage [5]. Cells involved in the bone repair process originate from bone marrow cells around the injured region [6]. We previously clarified that the numbers of bone marrow hematopoietic stem/progenitor cells (HSPCs) and mesenchymal stem cells (MSCs) decreased and increased in the injured region, respectively, 2 days after femoral injury in mice [7], and demonstrated that stromal cell-derived factor-1 (SDF-1) is involved in changes in the number of bone marrow stem cells in the bone repair process. Furthermore, we found that a fibrinolytic factor, plasminogen (Plg), and transforming growth factor-β (TGF-β) are involved in SDF-1 expression in this process [8].

**Fig. 1:**
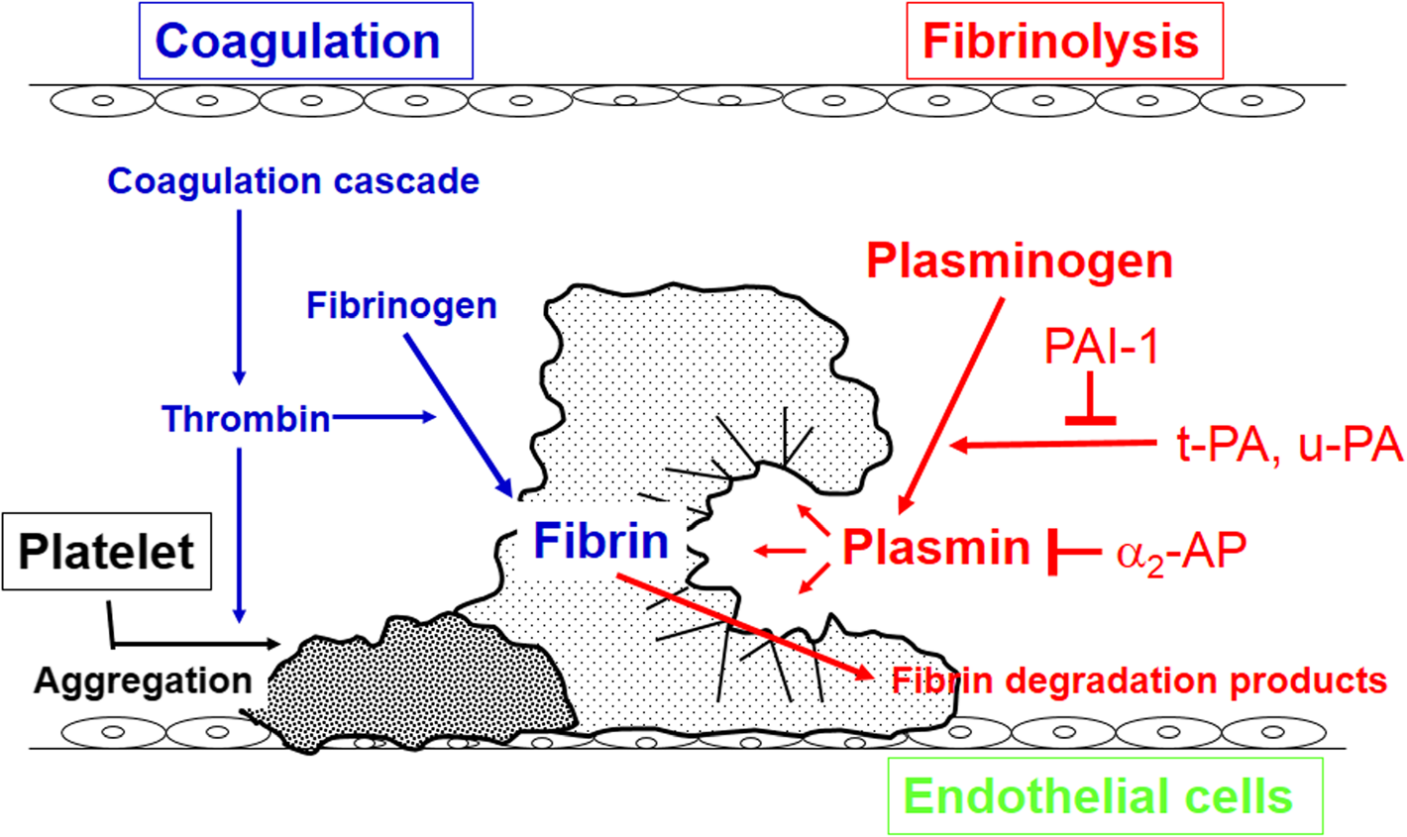
Fibrinolytic system in hemostasis. Plasminogen is converted by plasminogen activators (tissue-plasminogen activator, t-PA; urokinase-type plasminogen activator, u-PA) to enzymatically active plasmin on the surface fibrin which degrades fibrin, the main component of a thrombus, to fibrin degradation products (FDP). Plasmin is inactivated by its specific inhibitor, α_2_-antiplasmin (α_2_-AP), while plasminogen activators are inactivated by plasminogen activator inhibitor 1 (PAI-1)

In the fibrinolytic system, a serine enzyme zymogen, Plg, is converted to the active form, plasmin, and induces fibrin clot lysis [9] in hemostasic process, as shown in Fig. 1. Two types of plasminogen activators (PAs), urokinase-type PA (u-PA) and tissue-PA (t-PA), function in the conversion of Plg to plasmin. PA inhibitor, plasminogen activator inhivitor-1 (PAI-1), and the plasmin inhibitor α_2_-antiplasmin (α_2_-AP) are present as fibrinolytic system-controlling factors [9]. The fibrinolytic system plays diverse physiological and pathophysiological roles in addition to fibrin clot lysis [10]. Plasmin degrades the extracellular matrix directly or via MMP activation, through which growth factors present in the extracellular matrix, such as vascular endothelial cell growth factor (VEGF) and transforming growth factor-β (TGF-β), are released. In addition, the fibrinolytic system has diverse actions, including the activation of growth factors and intracellular signaling via the u-PA receptor (u-PAR) and protease activation receptors (PARs) expressed on the cell membrane, aiding in remodeling, repair/regeneration, inflammation, cell migration, and vascularization in many tissues [10–14].

In this report, the role of fibrinolytic factors in the bone metabolism/remodeling process and bone marrow stem cell induction process repair after bone injury is reviewed.

### Roles of fibrinolytic factors in the regulation of bone metabolism

Bone remodeling by osteoclasts and osteoblasts takes place in bone tissue and maintains the homeostasis of bone formation [1]. Gene deficient mice experiments suggested that fibrinolytic factors influence bone metabolism. In mice lacking both t-PA and u-PA, the bone mass increased and bone matrix protein accumulated, suggesting that PAs function in bone remodeling by degrading bone matrix protein by activating plasmin in vivo [15].

In Plg gene deficient mice, bone mineral densities of cortical and cancellous bone decreased, for which involvement of an increase in bone resorption through the promotion of osteoclast formation has been suggested [16]. Osteoclasts originate from monocyte/macrophage system cells. As it has been suggested that the Plg receptor is expressed on monocyte system cells and functions in cellular activities, such as cytokine production, Plg may influence bone metabolism via monocyte system cells in addition to being activated to plasmin by PAs and degrading bone matrix protein in mice.

### Roles of fibrinolytic factors in osteoclasts and osteoblasts

Osteoclasts originate from the monocyte/macrophage system and are involved in bone resorption [17]. Osteoclasts express t-PA, u-PA, PAI-1, and u-PAR [18]. Analysis of osteoclast differentiation from bone marrow cells suggested that the PAs/Plg system is not essential for osteoclast formation or bone mineral resorption, but it is involved in the degradation of noncollagenous bone matrix proteins [19]. Analysis of bone tissue collected from mice lacking both t-PA and u-PA suggested that PAs play a role in the adhesion of osteoclasts to bone tissues in the early stage of the bone resorption process [20]. On the other hand, u-PAR has been reported to function in osteoclast differentiation through an increase in macrophage colony stimulating factor (M-CSF) secretion from osteoblasts and activation of the phosphatidylinositol3-kinase/Akt/NF-κB pathway [21].

Osteoblasts originate from mesenchymal stem cells and are in charge of bone formation [22]. Osteoblasts express t-PA, u-PA, PAI-1, and u-PAR [23, 24]. It has been reported that PAI-1 expression by osteoblasts decreases in response to stimulation with parathyroid hormone, increasing PAs activity [23]. Bone mass is increased in t-PA and u-PA double-deficient mice [19]. On the other hand, Plg gene deficient mice-derived osteoblasts increased bone differentiation factor expression and promoted mineralization [25]. In addition, in a primary osteoblast culture system, the addition of active PAI-1 reduced gene expression of a bone differentiation markers and mineralization in mice [26]. Moreover, these effects by stimulation with active PAI-1 were observed in female mice-derived osteoblasts, suggesting the presence of sex difference in mice [26]. We investigated sex differences in the phenotypes of primary osteoblasts from male and female mice by using comparative comprehensive DNA microarray analyses. We identified a novel serine-protease inhibitor encoded by Serpina3n, the sex-specific factors in osteoblast that regulate bone metabolism in mice [27]. Moreover, we investigated the roles of PAI-1 in the differentiation of MSCs into osteoblastic cells using bone marrow or adipose tissue-derived MSCs from WT and PAI-1 gene deficient mice. We demonstrated that PAI-1 deficiency suppresses the differentiation of MSCs into osteoblasts in mice. PAI-1 might be a crucial factor for early stage osteoblastic differentiation of MSCs [28]. In u-PAR gene deficient mice-derived osteoblasts, the promotion of proliferating ability, increased ALP activity, promotion of mineralization, and increased bone matrix protein expression level were observed, suggesting inhibitory regulation of these osteoblast functions by u-PAR [24]. Furthermore, u-PAR has been reported to influence cell functions, such as osteoblast differentiation, via ERK1/2 and AP-1 [24]. It has also been suggested to induce osteoblast differentiation via complement C5a receptor expression on MSCs, leading to vascular calcification [29].

### Roles of fibrinolytic factors in the bone repair process

The tissue repair process is roughly divided into the acute inflammatory, repair, and remodeling stages [5]. Fibrinolytic factors have been suggested to function in repair/regeneration in the liver and skin tissues in mice [12, 13]. We investigated the role of fibrinolytic factors in the bone repair process using a fibrinolytic factor gene deficient mouse model with femoral defect. First, we reported that delayed bone repair accompanied by decreased cartilage and bone formation, and a decrease in the number of osteoblasts occurred after the bone became defective in the Plg gene deficient mice [25]. Moreover, Plg deficiency reduced vascular formation and VEGF and TGF-β expression levels, which are important for vascular formation, in the bone repair region. Furthermore, macrophage accumulation in the bone repair region decreased in the absence of Plg and TGF-β gene expression decreased in macrophages isolated from the bone marrow. These findings suggested that Plg functions in bone repair by accumulating macrophages in the bone repair region, and promoting subsequent TGF-β- and VEGF-associated vascular formation in mice.

In the u-PA gene deficient mice, early-stage bone repair was delayed, and macrophage accumulation and phagocytic activity in the bone repair region decreased [30]. In addition, the u-PA/Plg system was suggested to be involved in macrophage accumulation and activation via a chemokine, CCL3 in mice. In contrast, in the t-PA gene deficient mice, delay was noted in late-stage bone repair and t-PA was suggested to function in osteoblast proliferation in the bone repair region [31]. Furthermore, t-PA was suggested to promote osteoblast proliferation by activating ERK1/2 via annexin 2 in osteoblasts [31]. T-PA was also suggested to promote bone repair by increasing vascular formation through HIF-1α and VEGF expression in the bone defective region in mice.

### Roles of PAI-1 in diabetes-induced delayed bone repair

In diabetes, the fracture risk increases and bone repair is delayed. Using female mice of a streptozotocin (STZ)-induced diabetes model, we demonstrated that PAI-1 causes bone loss in the pathology of diabetes [32]. In addition, in a diabetic mouse femoral bone defect model in PAI-1 gene deficient mice, bone formation and recovery from reduction of the number of osteoblasts and delayed bone repair were observed in the diabetes-induced bone defective region [32]. Furthermore, PAI-1 deficiency inhibited the diabetes-induced reduction of bone differentiation markers. Based on these findings, PAI-1 functions in diabetes-induced delayed bone repair, and as a mechanism, reduction of the number of osteoblasts and inhibition of osteoblast differentiation in mice were suggested.

Furthermore, we demonstrated that STZ-induced diabetes mice decreases accumulation and phagocytosis of macrophages at the damaged site during early bone repair after femoral bone injury through PAI-1 in female mice [33].

### Roles of fibrinolytic factors in fracture healing

The roles of fibrin formation and fibrinolysis in fracture healing were investigated using fibrinogen and Plg gene deficient mice in fracture healing model [34]. Fibrinogen deficiency did not influence fracture healing, but in the Plg gene deficient mice, fibrin deposited in the fracture region remained, and vascularization and bone union were inhibited, delaying fracture healing. This suggested that fibrin is not essential for fracture healing, whereas fibrinolysis in the fracture region by plasmin is essential for fracture healing in mice. In the u-PA gene deficient mice, remodeling by osteoclasts of callus formed in the fracture healing process was inhibited [35]. In the PAI-1 gene deficient mice, enlargement of callus in the fracture healing process and subsequent rapid reduction of the callus were noted [36]. These findings suggested that u-PA and PAI-1 function in callus remodeling in the fracture healing process in mice.

### Role of SDF-1 in changes in bone marrow stem cells in the repair process after bone injury

Osteoblasts, osteoclasts, chondrocytes, and macrophages involved in the bone repair process originate from HSPCs and MSCs in the bone marrow [6]. Therefore, stem cells in the bone marrow may be strongly influenced by bone injury.

**Fig. 2:**
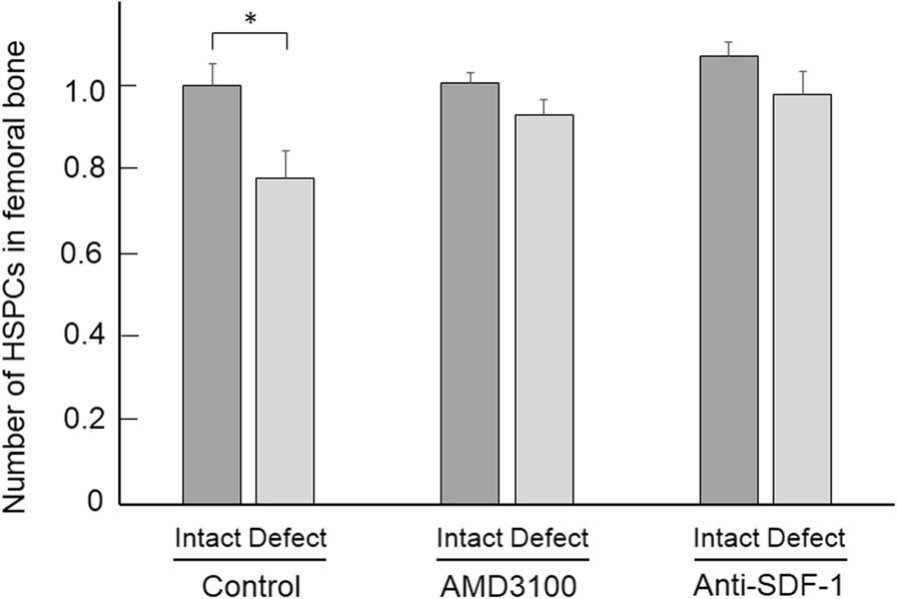
Effects of AMD3100 or anti-SDF-1 antibody on the number of HSPCs in the bone marrow after femoral bone damage in mice. The numbers of HSPCs in the bone marrow of the contralateral intact (intact) and damaged (defect) femurs 2 days after femoral bone damage in mice with or without intraperitoneal administration of AMD3100 (5 mg/kg) or local treatment with an anti-SDF-1 antibody (25 μg/body) were enumerated by flow cytometry. The data were expressed as the ratio of HSPC number to that in intact femurs of control mice [7]. The value of vertical axis is 1.0 = 2930 HSPCs/5 × 10^6^ BMCs in contralateral intact femurs in control mice. Data represent the mean ± SEM of 5 mice. *p < 0.05

We investigated changes in bone marrow stem cells in the bone repair process using a mouse femoral injury model [7]. The numbers of femoral HSPCs and MSCs on the injured side significantly decreased and increased, respectively, but no change was noted in the number of stem cells in the spleen after bone injury in mice. On the other hand, SDF-1 expression significantly increased in this injured bone region, and intraperitoneal administration of SDF-1-neutralizing antibody and local administration of SDF-1 receptor (CXCR4) antagonist AMD3100 inhibited bone injury-induced changes in the number of bone marrow stem cells in mice (Fig. 2) [7].

The chemokine SDF-1 plays an important role in maintaining the HSPC localization and cell cycle status in the bone marrow. Chemokines are chemotactic cytokines forming a chemical gradient for cell migration. SDF-1 and its receptor, CXCR4, are involved in bone marrow cell migration and survival in mice [37]. SDF-1 is also involved in cell migration in the fracture region [38] and SDF-1/CXCR4 signal transduction is important for repair after fracture in mice [39].

These findings revealed that a decrease in the number of HSPCs and increase in the number of MSCs in the repair process after femoral injury occur in parallel in the bone marrow on the injured side. In addition, it was suggested that the SDF-1/CXCR4 system is involved in changes in the stem cell population after bone injury in the bone marrow in mice [7].

On the other hand, female mice of a STZ-induced diabetes model attenuated a decrease in the number of HSPCs in bone marrow induced by bone injury in mice [33].

### Roles of fibrinolytic factors in changes in bone marrow stem cells in the repair process after bone injury

We previously reported the involvement of fibrinolytic factors in the repair process after bone injury using gene deficient mice of fibrinolytic factors, as described above. In the Plg gene deficient mice, impairment of macrophage accumulation in the injured region and delayed bone repair accompanied by the inhibition of vascularization occur in the repair process after bone injury [25]. As macrophages originate from HSPCs, Plg may influence HSPCs in the repair process after bone injury in mice.

**Fig. 3:**
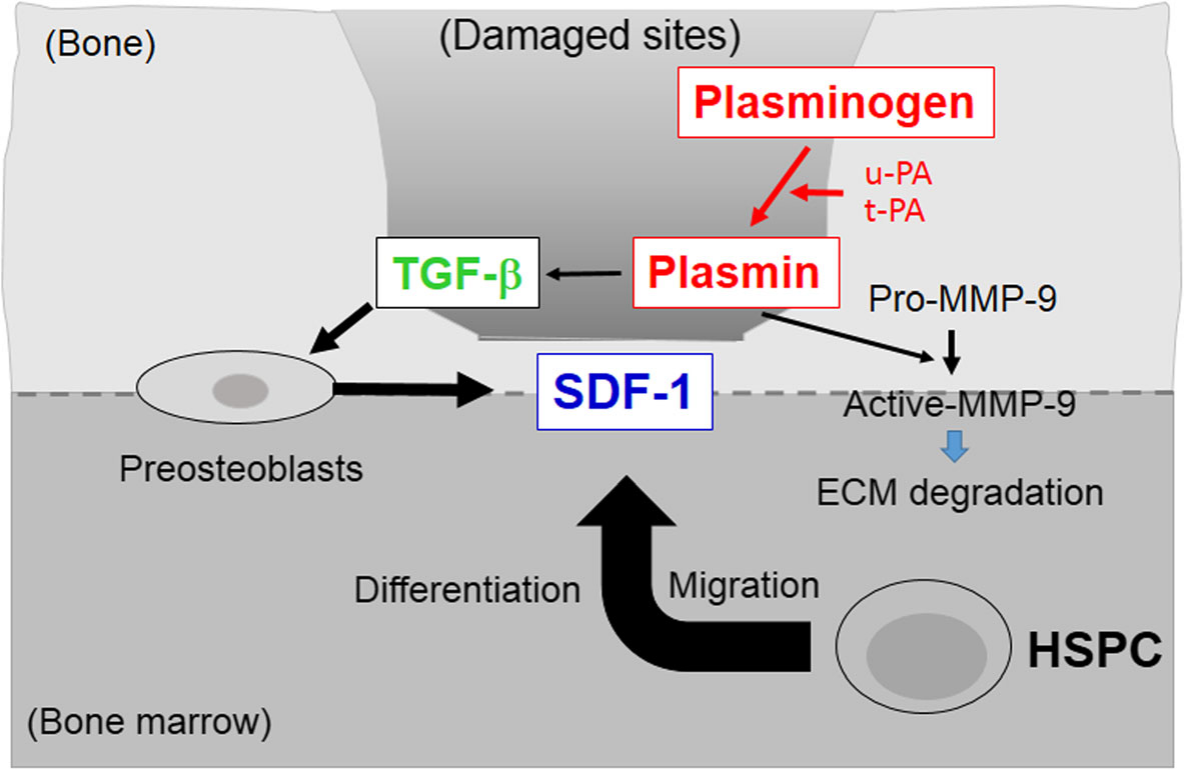
Roles of fibrinolytic factors in bone marrow stem cell changes during the repair process after bone injury. Plg (plasmin) induces the production and release of TGF-β from the extracellular matrix via MMP-9 activation after bone injury. SDF-1, expressed by endosteal preosteoblasts, induces the reduction and mobilization of bone marrow HSCs

We investigated changes in bone marrow stem cells in the repair process after bone injury in Plg gene deficient mice [8]. Plg deficiency significantly slowed the reduction of the number of HSPCs induced after bone injury, but it did not influence the increase in the number of MSCs in mice. In addition, Plg deficiency significantly slowed the increase in the number of cells positive for both bone injury-induced endosteal SDF-1 and Osterix in mice. Furthermore, it significantly slowed bone injury-induced increases in SDF-1 and TGF-β expression in mice. These reactions after bone injury in the Plg gene deficient mice were resolved by administration of a TGF-β signal inhibitor. The above findings suggested that Plg is closely involved in the induction of bone marrow HSPCs in the repair process after bone injury through TGF-β and SDF-1 in mice. Based on the results of cells positive for both SDF-1 and Osterix, SDF-1 expression induced after bone injury may originate from preosteoblasts present in the endosteum in mice.

u-PA activity, t-PA activity, and activated MMP-9 activity were increased in the injured region in the repair process after bone injury, but in the Plg gene deficient mice, the increase in bone injury-induced PA activity and activated MMP-9 activity was slowed [8]. The niche microenvironment is involved in the proliferation and differentiation of HSPCs in the bone marrow [40]. Plg has been suggested to be important for mouse granulocyte colony stimulating factor (G-CSF)-induced HSPC mobilization and hematopoietic regeneration [41, 42], and plasmin activates pro-type MMPs such as MMP-9. MMP-9 is associated with the extracellular degradation of matrix, and subsequent tissue repair and regeneration process in mice [43]. Plg responds to G-CSF through the activation of mouse MMP-9 and regulates SDF-1-mediated HSPC mobilization [42]. We previously showed that Plg deficiency suppresses MMP-9 activity at the bone injury site after a femoral bone defect in mice [8]. In that study, the bone injury enhanced MMP-9 activity at the damaged site and its maximal effects were observed when the bone marrow HSCs population changes were observed. These findings suggest that MMP-9 might be a downstream factor of the fibrinolytic system-induced changes in HSPCs in the bone repair process after bone injury in mice. In addition, TGF-β-producing cells in the repair process after bone injury may be macrophages [44, 45]. We previously suggested that Plg is important for TGF-β induction in macrophages in the injured region after femoral injury in mice [25]. Based on the above, as shown in Fig. 3, Plg (plasmin) is considered to induce TGF-β production in macrophages involved in the bone repair process and induce TGF-β release from the extracellular matrix via MMP-9 activation after bone injury in mice [8]. On the other hand, SDF-1 is expressed by endosteal preosteoblasts, and induces the reduction and migration of bone marrow HSPCs in mice.

## Conclusions

Fibrinolytic factors play an important role in the regulation of bone metabolism and the bone repair process. Changes in fibrinolytic factors influence osteoblasts and osteoclasts directly or indirectly, and the collapse of normal bone remodeling may cause bone metabolism abnormalities such as osteoporosis. Indeed, in ovariectomized mice, an animal model of postmenopausal osteoporosis accounting for the majority of osteoporosis cases, PAI-1 and α_2_-AP function in the ovariectomy-induced decrease in bone mass density [46, 47]. In addition, investigation using knockout mice demonstrated that PAI-1 plays a role in steroid-induced osteoporosis, which is the most important secondary osteoporosis, and diabetes-associated osteoporosis in mice [48]. Involvement of bone marrow HSPCs and MSCs in the early stage of the repair process in mice has also been suggested [7, 8]. However, the relationship between fibrinolytic factors and bone marrow stem cells during bone repair remains unclear. Elucidation of the detailed mechanism underlying the influence of fibrinolytic factors on bone metabolism and bone repair, and progression of related clinical studies are expected to lead to the establishment of a fibrinolytic factor-targeting method to efficiently promote bone repair/regeneration and fracture healing, and development of a new osteoporosis treatment method and diagnostic marker.

## Data Availability

Not applicable.

## Abbreviations

α_2_-AP: α_2_-antiplasmin
CCL3: C-C motif chemokine ligand 3
G-CSF: Granulocyte colony stimulating factor
HSPC: Hematopoietic stem/progenitor cell
M-CSF: Macrophage colony stimulating factor
MMP: Matrix metalloproteinase
MMP-9: Matrix metalloproteinase-9
MSC: Mesenchymal stem cell
Plg: Plasminogen
PA: Plasminogen activator
PAI-1: Plasminogen activator inhivitor-1
STZ: Streptozotocin
SDF-1: Stromal cell-derived factor-1 receptor
CXCR4: C-X-C chemokine receptor type 4
t-PA: Tissue-plasminogen activator
TGF-β: Transforming growth factor-β
u-PA: Urokinase-type plasminogen activator
u-PAR: Urokinase-type plasminogen activator receptor
VEGF: Vascular endothelial cell growth factor
